# The Effect of the Modified Eighth Section of Eight-Section Brocade on Osteoporosis in Postmenopausal Women: A Prospective Randomized Trial

**DOI:** 10.1097/MD.0000000000000991

**Published:** 2015-06-26

**Authors:** Bao-Xin Liu, Shu-Peng Chen, Yu-Dong Li, Ji Wang, Bin Zhang, Ying Lin, Jun-Hui Guan, Ying-Feng Cai, Zhu Liang, Fang Zheng

**Affiliations:** From China Department of Orthopedic, Guangzhou Hospital of Traditional Chinese Medicine, Guangzhou, China (BXL, BZ, JHG, YFC); The State Key Laboratory of Oncology in South China, Cancer Center, Sun Yat-sen University, Guangzhou, China (SPC); Department of Orthopedic, Liuzhou Traditional Chinese Medical Hospital, Third Affiliated Hospital, Guangxi Traditional Chinese Medical College, Guangxi, China (JW, ZL); State Key Laboratory of Ophthalmology, Zhongshan Ophthalmic Center, Sun Yat-sen University, Guangzhou, China (YL); and Guangdong Provincial Key Laboratory of Malignant Tumor Epigenetics and Gene Regulation, Medical Research Center, Sun Yat-Sen Memorial Hospital, Sun Yat-sen University, Guangzhou (FZ, LYD).

## Abstract

Osteoporosis and related fragility fractures represent a serious and global public health problem.

To evaluate whether the modified eighth section of Eight-section Brocade (MESE) exercise could improve the symptom and indexes associated with osteoporosis in postmenopausal women.

Guangzhou and Liuzhou hospital of traditional Chinese medicine in China.

Women (n = 198) aged 50 to 75 years were randomized into Control, Ca, MESE, and MESE + Ca.

Subjects in Ca and MESE groups were separately asked to consume thrice daily Calcium Carbonate Chewable D3 tablet and to perform thrice daily MESE exercise by 7 repetitions per time for 12 months. Subjects in MESE + Ca group performed such the combined treatment project for 12 months. Body height and Hospital for Special Surgery (HSS) scores of both knees, chronic back pain visual analogue scale scores (VAS), bone mineral density (BMD) at L2 to L4 and the left femoral neck, 3-feet Up and Go Test (3′) and one-leg Stance (OLS).

In our study, the improvement in chronic back pain of the patients in Ca, MESE, and MESE + Ca group was better than that in control group. There was 1.9% and 1.7%, 2.3%, and 2.1% net profit in left femoral neck and lumbar BMD after the treatment for 12 months in MESE and MESE + Ca groups. For the balance capacity, the subjects in MESE and MESE + Ca groups secured much better performance than those in Ca and control group after the treatment for 12 months (*P* < 0.001, *P* < 0.001).

The treatment of MESE exercise is the most effective for the improvement of the symptom and indexes in postmenopausal women. Importantly, the low attrition and the high exercise compliance indicate that MESE exercise is safe, feasible, and well tolerated by postmenopausal women.

## INTRODUCTION

Osteoporosis and related fragility fractures represent a serious and global public health problem that is projected to increase with the aging of population.^1^ Currently, it is estimated that 1 in 3 women aged over 60 years will suffer an osteoporotic fracture in their lifetime.^2^ The dominant factor affecting bone metabolism in postmenopausal women is the decline in estrogen secretion although other factors such as decreased physical activity have been identified.^3,4^ Hormone replacement therapy (HRT) was the main method to prevent osteoporosis or climacteric symptoms. Unfortunately, hormonal replacement therapies may have serious cardiac, vascular, hepatic adverse effects and may have an ominous impact on carcinogenesis and endometrial.^5^ So it is urgent to identify safe, inexpensive, and widely accessible evidence-based strategies to prevent and manage osteoporosis.

Exercise and calcium-vitamin D(Ca) are recognized as key modifiable lifestyle factors that have been shown to be important for the prevention and management of osteoporosis, particularly in older women.^6^ The current authors reported that low-frequency (12.6 Hz and 3 mm of amplitude) vibratory exercise using a reciprocating plate is feasible and is effective to improve 2 major determinants of bone fractures in postmenopausal women: hip bone mineral density (BMD) and balance.^7^ It has been shown by a number of randomized controlled trials conducted in postmenopausal women that exercise, particularly high intensity progressive resistance training (PRT) and/or weight-bearing impact exercise, which is supplemented with calcium and/or vitamin D, can yield small profits (1–3%), or decrease age-related losses in BMD at the hip and spine.^8–10^ The mechanism of effect of exercise and Ca on BMD are different maybe because the benefit of exercise is in the site-specific modification while Ca produces the permissive systemic influence.^11,12^ But some studies have documented that the skeletal gains of exercise may depend on adequate dietary calcium intakes (>1000 mg/day).^13^

Eight-section Brocade which is composed of 8 sectional different actions is a traditional and safe physical therapy that is widely accepted in the treatment of the disease, rehabilitation, and sports medicine. The first 7 sections in Eight-section Brocade have been mainly associated with the twisting action of the body. However, the eighth stance, which is also known as “Jolt Body to Keep All Illnesses Away,” “Lift the Back,” and “Cure many Illnesses,” may stimulate the immune system and enhance the power of osteogenesis by the perpendicular stress resulting from the body gravity. According to the Chinese medicine, belonging to the urinary bladder, urinary Bladder Channel of Foot-Taiyang, which is attached directly to unusual internal organ, such as the heart and the brain, may take in and transmit the primordial essence into Wu Zang in order to cooperate each other in physiology. Starting at the inner canthus, urinary Bladder Channel of Foot-Taiyang goes up to the vertex of the head. Its straightforward channel goes from the vertex into the skull to connect with the brain before returning downward and branching off at the nape. Then it goes along both sides of the vertebral column to the waist, connects with the kidney, and belongs to the urinary bladder. It goes further downward, through the buttocks, to enter the middle of the knee hollow. Its another branch goes downward from the internal margin of the shoulder blade and, through the buttocks, converges with the above-mentioned branch at the middle of the knee hollow. There it goes still downward, past the back of lower leg, along the lateral insept to the tip of the small toe. At the same time, the eighth gesture may relieve pain by promoting the physiological activities of urinary Bladder Channel of Foot-Taiyang, and clearing up the channel in the lower extremity and lumbar spinal cord.^14^ However, there are no data on the effect of the Eight-section Brocade on the osteoporosis. The present study aimed to observe the effects of the modified eighth section of Eight-section Brocade (MESE) therapy on bone mass in postmenopausal women with osteoporosis.

## METHODS

### Ethical Issues and Setting

A total of 383 outpatients, including postmenopausal women, were studied from Liuzhou traditional Chinese Medical Hospital (221) and Guangzhou Hospital of Traditional Chinese Medicine (162) during the period of March 2009 to March 2013, with informed consent under institutional review board-approved protocols, which includes purposes and procedures of the study. The research was approved by the Institute Research Medical Ethics Committee of Sun Yat-Sen Memorial Hospital, Guangzhou, China.

### Inclusion Criteria

The BMD in the L2 to L4 and hip was identified in women with inadequate bone mass or osteoporosis, the diagnosis of which was based on the 1994 criteria of the World Health Organization. Spine, hip, spine, or wrist osteoporosis of women with BMD < –2.5 SD (T score < −2.5) should be defined, compared to normal healthy young women. And the postmenopausal women without the traumatic fracture in any sites and typical menopausal symptoms would like to participate in this experiment as volunteers, aged from 50 to 75 years.

### Exclusion Criteria

Exclude were women with cardiovascular or cerebrovascular disease, women with blood pressure higher than 160/110 mmHg on medication, women with systolic blood pressure less than 90 mmHg, women with heart stents or body implantation, women with a history of thrombosis within the past 6 months, women unrehabilitated from surgical operation, women which have been treated with drugs for osteoporosis or other agents which affect bone metabolism, women unrecovered from muscle strain joint injuries or fractures, women with symptoms of vertigo and in poor health, women with spinal nerve canal stenosis, spondylolisthesis, or lumbar disc herniation, and women with epileptics.

### Grouping

**FIGURE 1:**
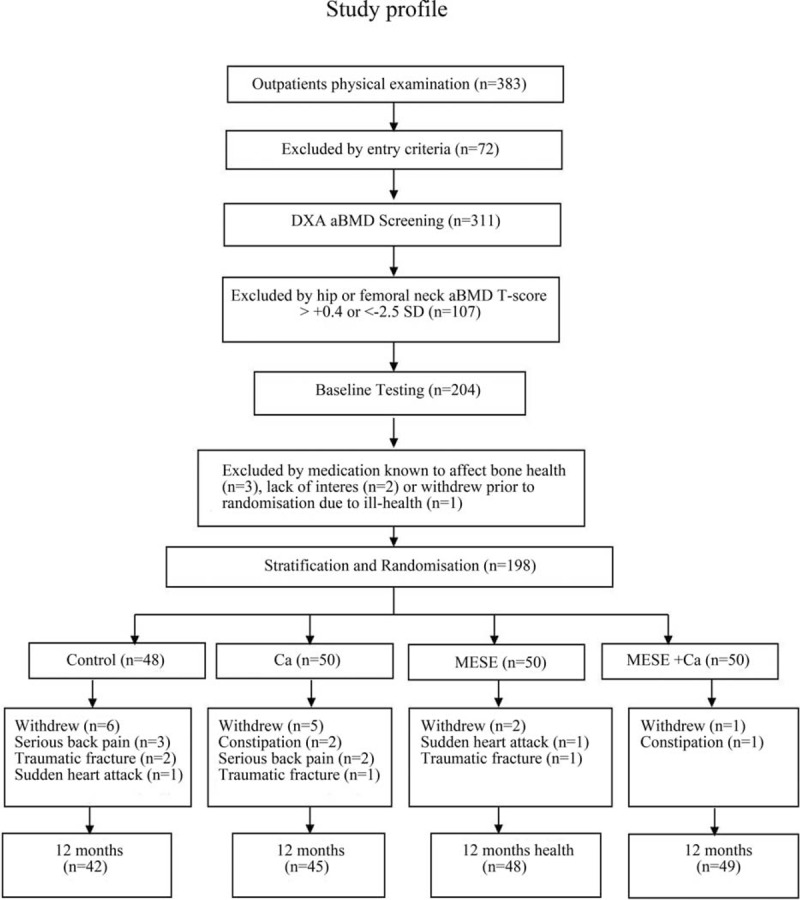
Study profile.

This 2 × 2 factorial design study was a 12-month randomized controlled trial. The 2 factors were MESE exercise and Ca, each tested on 2 levels so that the women who met the inclusion criteria and had no contraindications were randomly allocated to 1 of 4 groups, a control group (n = 48); Ca alone (n = 50); MESE exercise alone (n = 50); MESE + Ca (n = 50) (Figure 1). Of the 198 women, 184 completed the 12-month experiment. In control group, 6 were lost, because of serious back pain (n = 3), traumatic fracture (n = 2), and sudden heart attack (n = 1). In Ca group, 45 completed the experiment and 5 lost, including 2 lost the treatment of constipation, 2 was due to medication for serious back pain, the others left one failed to traumatic fracture. In MESE group, 48 women completed and 2 defaulted on the experiment, including one lost the treatment of sudden heart attack, the other were due to traumatic fracture. In MESE + Ca group, only 1 defaulted on the experiment for constipation.

### Treatment Methods

Participants in Ca group (n = 50) were asked to consume Calcium Carbonate Chewable D3 tablet (500 mg of calcium and 200 IU of vitamin D3, thrice daily) (GE Healthcare, Shanghai, China) for 12 months. Patients in MESE group (n = 50) were given exercise of MESE by 7 repetitions per time, thrice daily. Subjects in MESE + Ca group (n = 50) performed such the combined treatment project for 12 months.

### Test Indicators

Chronic back pain was evaluated by visual analogue scale (VAS) at the baseline, the 3rd week, the 6th month, and the 12th month of the treatment. The BMD at L2 to L4 and the left femoral neck of all subjects was determined before the start of the treatment and at the 12th month of treatment respectively (dual-energy bone densitometers, M2428, the Medlink Corporation, France).

### The Modified Eighth Section of Eight-Section Brocade (MESE)

The modified eighth section of Eight-Section Brocade also called “jolt the back 7 times” is introduced below.

Stand up straight (the older or the weak may lean on the wall). The toes should be pointed straight ahead, with the feet parallelly apart to one's shoulders width and one's knees slightly bent to 135∼170°. One's arms should hang down in a relaxed manner at one's sides. Relax one's body for 2 minutes. Stay balanced and centered. Clear one's mind and set aside the work and worries of the day. One's face should seem happy, joyful, reflecting an “Inner Smile.” Keep one's head up and look forward. One's eyes should be open, with a soft and wide angle focus. Breathe in and out in a relaxed, easy, and regular manner. Keep one's lips parted slightly. Next, raising slowly 2 arms and one's heels to the highest with the palm downwards as you slowly deeply inhale. Then, both one's arms and heels fall under gravity after holding one's breath for 5 to 10 seconds. Finally, the waist lightly vibrates as soon as one's heels downcasts to touch the ground. Subsequently, one's legs slowly squats down and one's knees slightly bent to 135∼170° with exhaling slowly deeply. Repeat steps above. Work up to get up to 7 repetitions.

### Bone and Body Composition

BMD was measured by dual-energy x-ray absorptiometry (DXA) at the lumbar spine (L2–L4) and left proximal femur on the nondominant side, using the standard protocol. All analyses have been carried out by the same investigation in order to minimize interobserved variation.

Body height was measured by weighing scales. Both knees function was measured by Hospital for Special Surgery (HSS) knee score.

### Balance and Motility Performance Measures

Participants were undertaken 2 kinds of balance tests: 3-feet Up and Go Test (3′UG) and one-leg Stance (OLS). At first, each subject remained seated and rested for 5 minutes before the test. The score of 3′UG test which were measured to the nearest one-tenth of a second, were recorded as the shortest time from the seated position, walking 3 ms, then turning, and returning to the starting point.

In OLS tests, each subject should stand up straight as possible as in a unipedal posture on the floor with the eye open, head upright, arms relaxed by the side of the body. The score was equivalent to the time from the moment 1 foot was raised from the ground to when it touched the floor or the standing leg. The longer time it stood, the better balance ability was. And the maximum time was 45 seconds. Participant could perform twice OLS tests, with the rest for 1 minute between 2 attempts. And the best performance was recorded as OLS score.

### Statistical Analysis

Statistical analyses were performed using SPSS 16.0 (SPSS, Inc., Chicago, IL). Baseline characteristics between the groups were compared using analysis of variance. The one-way ANOVA test was used for comparing groups, and the Bonferroni post hoc test was used for multiple comparisons when ANOVA was significant. Before and after treatment values of variables in each group were analyzed with paired *t*-test. Descriptive data were calculated for the study sample and presented as means ± standard deviation (SD). The smaller *P*-values than 0.05 are considered to be statistically significant.

## RESULTS

### Recruitment

Fourteen of 198 women (7.1%) withdrew from the study over the 12-month period (Control, n = 6; Ca, n = 5; MESE, n = 2; MESE + Ca, n = 1). The reasons for the withdrawal included: constipation related to the study (n = 3), the serious back pain (n = 5), the fracture (n = 4), and illness unrelated to the study (n = 2). The average compliance with the calcium D3 program was 90%, and did not differ between the MESE + Ca (98%) and Ca alone group. The average compliance with MESE was 96% and was no different between the MESE + Ca and MESE groups (Figure 1).

### Subject Characteristics

Demographics and descriptive parameters of the subjects are listed in Table 1. On average, participants suffered from the similar pain level, lumbar BMD, and left femoral neck. There were no significant between-group differences in baseline characteristics, including age, menopausal years, VAS, L-BMD, F-BMD, 3′UG, OLS, Height, and HSS among the four groups (all *P* > 0.05). The average dietary calcium intake for all participants was 1.5 g/day. And no participants had severe vitamin D deficiency.

**TABLE 1:**
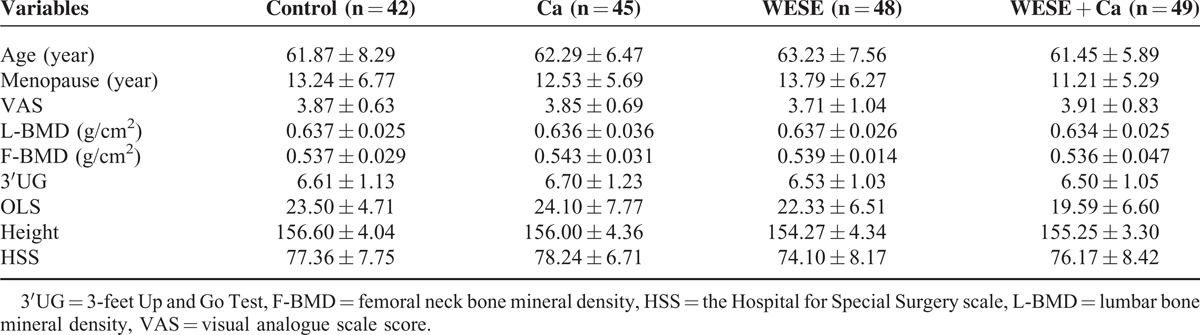
Baseline Characteristics of the 3 Groups (Mean ± SD)

### Compliance with Intervention and Adverse Events

There were no serious injuries or adverse events associated with exercises. The limited number of minor injuries included the exacerbation of the knee or hip pain (n = 2, both are able to continue with exercise after program modification), lower back injury (n = 1, 1 recovered with 1-week rest).

### Pain Visual Analogue Scale Scores

**FIGURE 2:**
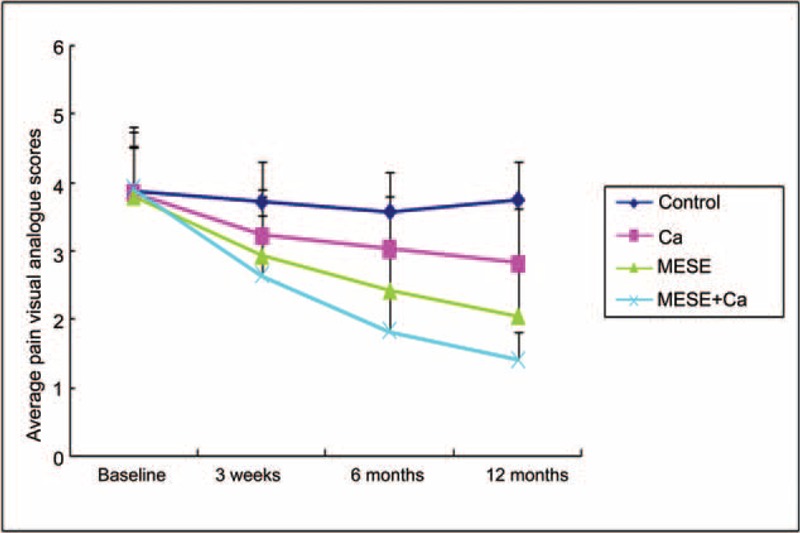
Average pain visual analogue scores in Control (n = 42), Ca (n = 45), MESE (n = 48), MESE + Ca (n = 49) groups at baseline and after the treatment for 3 weeks, 6 months, and 12 months. MESE exercise and MESE + Ca resulted in significant decreases in VAS relative to both baseline and control group. Data are presented as means ± SE (all *P* < 0.05, AVON).

After the treatment for 3 weeks, it has been more effective for Ca, MESE, and MESE + Ca to alleviate patient's back pain, compared to control group. But there has been no the significant difference for VAS in Ca group from 6 months to 12 months (*P* > 0.05). Within-group analysis revealed that VAS at each time point decreased significantly in MESE and MESE + Ca groups relative to control group. And the improvement in chronic back pain of the patients in group MESE + Ca and group MESE was better than that in group Ca after the treatment for 12 months (*P* < 0.05, *P* < 0.001; Figure 2). In addition, with the prolongation of the time, the VAS of chronic back pain of the patients in group MESE + Ca and group MESE was lower than that both in control and in Ca group (*P* < 0.001, *P* < 0.001). So it is effective for MESE exercise to alleviate patient's pain.

### Bone Mineral Density

**FIGURE 3:**
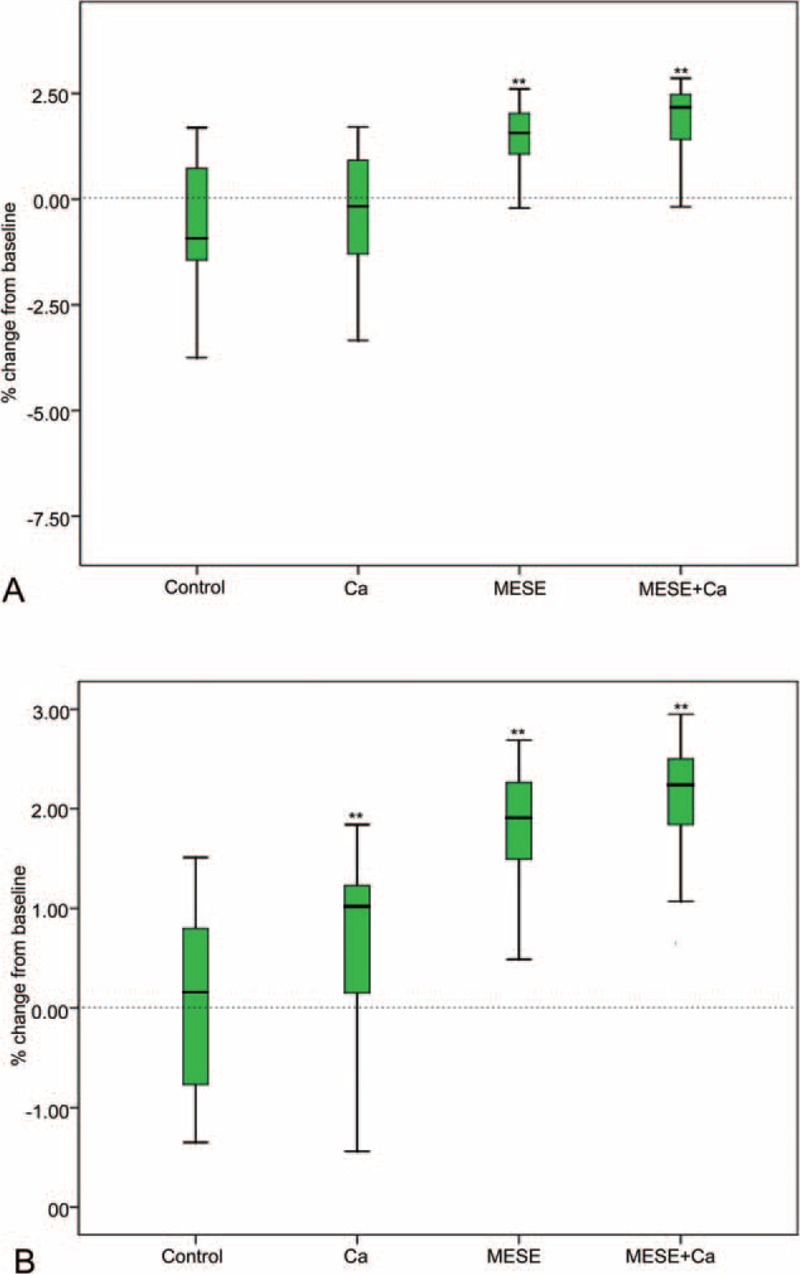
Mean adjusted percentage change (95% CI) in left femoral neck and lumbar BMD in Control (n = 42), Ca (n = 45), MESE (n = 48), MESE + Ca (n = 49) groups. ^∗∗^*P* < 0.001 within-group change from baseline. (A) Mean-adjusted percentage change (95% CI) in left femoral neck BMD in Control (n = 42), Ca (n = 45), MESE (n = 48), MESE + Ca (n = 49) groups. The increase in left femoral neck BMD in MESE and MESE + Ca groups was significantly greater than the increases in Ca group (*P* < 0.001). (B) Mean-adjusted percentage change (95% CI) in lumbar BMD in Control (n = 42), Ca (n = 45), MESE (n = 48), MESE + Ca (n = 49) groups. At lumbar L2 to L4 BMD, there was a significant main effect of MESE exercise (*P* < 0.001).

BMD measured by DXA offers sufficient precision to follow disease progression and is currently the best-validated technology for showing a response to therapy. So the effect of Ca, MESE exercise, MESE + Ca on left femoral neck and lumbar BMD in postmenopausal women with osteoporosis were assessed after the treatment for 12 months. MESE resulted in a significant 1.9% net gain in left femural neck BMD relative to control after 12 months (*P* < 0.001; Figure 3A). Similar results were observed in MESE + Ca groups (2.3%). For left femural neck BMD, there was a significant 1.4% to 1.9% within-group increase in both exercise groups. But the BMD in MESE + Ca group significantly increased than BMD in MESE group (*P* < 0.05; Figure 3A). Maybe MESE exercise facilitated the absorption of Calcium in human body. Comparable results were observed for lumbar spine (L2–L4) BMD, and there was a significant net 1.7% to 2.1% increase in BMD in all 3 treatment groups compared to the control group. In the subsequent per protocol analysis, the exercise main effects remained significant at lumbar BMD in MESE and MESE + Ca groups (*P* < 0.001, *P* < 0.001; Figure 3B), and between-group differences in favor of the exercise group were also detected at lumbar spine (L2–L4) (*P* < 0.001; Figure 3B). And there was no the significant difference about the lumbar BMD in both MESE and MESE + Ca groups after the treatment for 12 months (*P* > 0.05; Figure 3B). This is likely due to the significant 1.0% within-group increase in lumbar BMD in Ca alone group. So MESE exercise resulted in the main effect on BMD.

### Change in Balance Capacity

**FIGURE 4:**
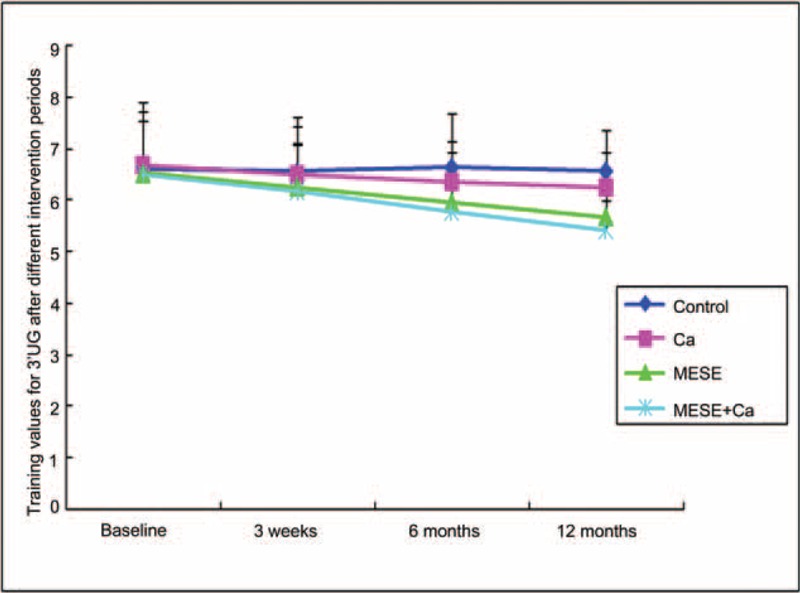
Training values for 3′UG in Control (n = 42), Ca (n = 45), MESE (n = 48), MESE + Ca (n = 49) groups after the different intervention periods. With the prolongation of the time, MESE exercise and MESE + Ca treatment have significantly decreased training values for 3′UG, compared with the baseline and control (*P* < 0.001, *P* < 0.001). Data are presented as means ± SE (all *P* < 0.05, AVON).

After the treatment for 3 weeks, we observed that there were no significant difference for 3′UG test in Ca, MESE, and MESE + Ca groups, compared to control group (*P* > 0.05; Figure 4). But the subjects in MESE and MESE + Ca groups secured much better performance than those in control group after the treatment for 6 to 12 months (*P* < 0.001, *P* < 0.001; Figure 4). And there was no significant difference between Ca and control groups (*P* > 0.05; Figure 4). And subjects in MESE and MESE + Ca groups made the greater improvement by 0.87 and 1.17 seconds decrease in 3′UG test, relative to ones in control group (Figure 4). Meanwhile, it has been illustrated in Figure 4 that there was a significant 0.57 and 0.87 seconds decrease for 3′UG in MESE and MESE + Ca groups, compared to that in the Ca group.

**FIGURE 5:**
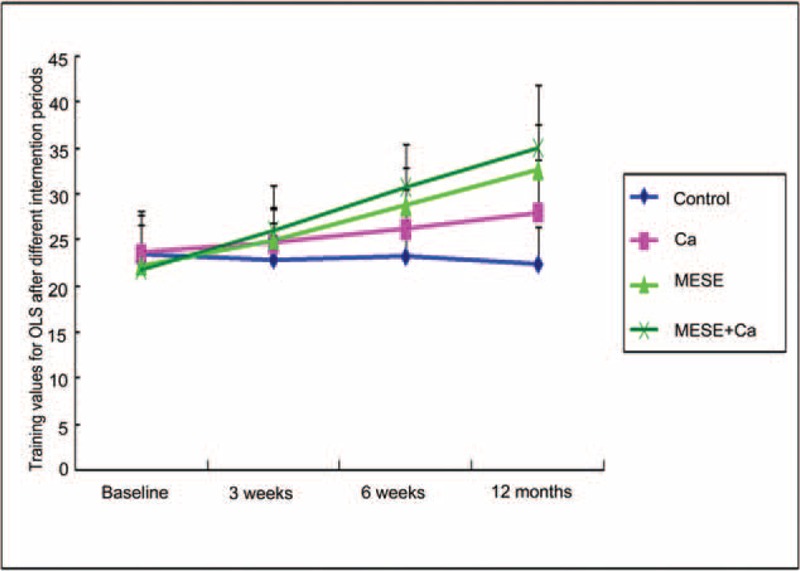
Training values for OLS in Control (n = 42), Ca (n = 45), MESE (n = 48), MESE + Ca (n = 49) groups after the different intervention periods. MESE exercise and MESE + Ca resulted in the increase of the length of participation in OLS, compared with the baseline and control (*P* < 0.001, *P* < 0.001). Data are presented as means ± SE (all *P* < 0.05, AVON).

In OLS test, the length of participation in Ca, MESE, and MESE + Ca therapy was significantly longer 1.75, 2.23, and 3.21 seconds than control group after the treatment for 3 weeks (*P* < 0.05, *P* < 0.001, *P* < 0.001; Figure 5). All the more, there was a significant 0.48 and 1.46 seconds increase in MESE and MESE + Ca group, compared to Ca group (*P* < 0.05, *P* < 0.05, *P* < 0.05; Figure 5). And with the prolongation of medication, the more evident was it for MESE exercise to improve the balance capacity (*P* < 0.001; Figures 4 and 5). So at 12 months, the time for OLS test in MESE and MESE + Ca groups got up to 32.6 and 35.1 seconds (Figure 5). And there was the significant difference in 3′UG and OLS tests between MESE and MESE + Ca group after the treatment for 3 weeks to 12 months (*P* < 0.05, *P* < 0.05; Figures 4 and 5). At the same time, it has been demonstrated in Figure 4 that the performance of Ca group for 3′UG is not significantly changed by the treatment from calcium for 3 weeks to 12 months. So maybe the traditional calcium intakes only increased the time of OLS test, and MESE exercise may not only extend the time of OLS but also enhance the velocity of 3′UG.

### Body Height and HSS Scores of Both Knees

There were no difference existing among the body height and HSS scores of both knees in 3 groups, compared to those in control group. Table 2 shows the results for the body height and HSS scores after the treatment for 12 months. MESE and/or Ca cannot significantly enhance the body height and improve the HSS scores of both knees.

**TABLE 2:**
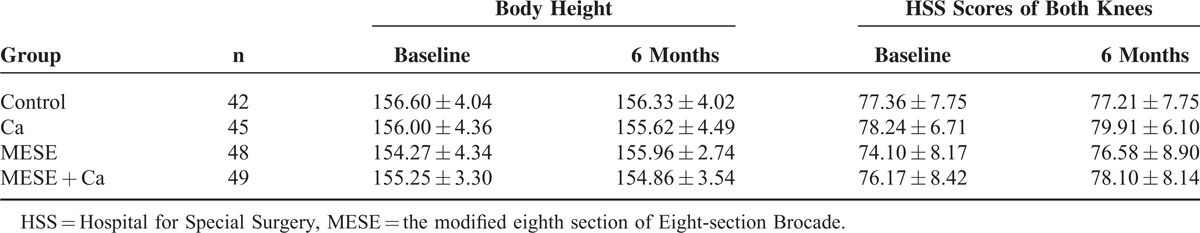
Comparison of Body Height and HSS Scores of Both Knees at Baseline and Follow-Up Periods

## DISCUSSION

The main findings of the present study were that the treatment of MESE exercise was the most effective for lowering VAS, increasing the left femoral neck and lumbar BMD and balance in posture, which are associated with the increased bone fragility and higher risk of fracture. However, we also observed that only supplementation with 1500 mg of calcium and 600 IU of vitamin D3 per day cannot enhance the 3′UG, the left femoral BMD, which is consistent with the earlier results.^2^ At the same time, there are no significant difference for the effect between MESE and MESE + Ca groups on the lumbar BMD. So it is very effective for MESE to meliorate the symptoms and indexes on osteoporosis.

In many studies, several lines of evidence have been shown that the exercise training plays the important role in the prevention of osteoporosis via development as well as maintenance of BMD.^15,16^ Based on the findings of a serial of basic animal research, the theory of practical exercise application has formed.^17,18^ Robling think that it is effective for an exercise regime to improve parameters of bone strength dependent on the known reactivity of bone cells and tissue to all kinds of load, which has been referred as the osteogenic index (OI).^19^ Although the prolonged training stimuli has desensitized animal bone tissues, the bone response to a cyclical stimulus could been markedly improved by adding rest periods between bouts of loading.^20^ And the bone response to training varies along with nature of the activity, diet, reproductive hormone status, and the different function of skeletal age. Certainly, the practical goal of an exercise intervention does not merely lie in increasing bone mass, but in reducing the incidence of fracture.^21^ Although it is very effective for highly intense and explosive exercise to stimulate the osteogenesis, this kind of training which has not been recommended for the older man or the patient with osteoporosis, not only impose an additional burden on the circulatory system, but also tend to result in the specific fracture. So the modified eighth section of eight-sectioned brocade has spontaneously complied with the principle of individual, such as the choice of the strength and length of participation in exercise based on their own specific condition of the older person, including the change of individual's bone mass, the degree of the seriousness, and the fracture.

The length of participation in weight-loading exercise may be an important consideration for exercise programming in older adults. No change in femoral neck bone mass was observed in postmenopausal women following 9 months of jump plus resistance exercise wearing weighted vests.^22^ A study on the associations of physical activity, body mass index and BMD in 6442 women found that 5 years of participation in physical activity is associated with a small increase in total hip BMD.^23^ In our study, the length of the time for the loading-weight, such as the posture of the squat and the tiptoe, was based on the individual's specific condition. And there was 1.9% and 1.7%, 2.3%, and 2.1% net profit in left femoral neck and lumbar BMD after the treatment for 12 months in MESE and MESE + Ca groups. Likewise, MESE exercise resulted in the decrease of the time in the 3′UG test, consistently, improved the balance capacity. It is possible that the delayed response is a function of mineralization, a process known to continue long after new bone tissue (osteoid) has been secreted by osteoblasts.

Exercise may obviously accelerate the blood circulation in the whole body and the skeleton. At the same time, the contraction and relaxation of the muscle may also stimulate the osteogenesis. In turn, those may prevent and decelerate the process of osteoporosis development. It has been commonly accepted that the participation in moderate physical activity stimulates the osteogenesis to the bones,^24^ by enhancing serum concentration of bone formation marker^25–29^ as well as bone resorption markers.^30–32^ Eight-section Brocade is a kind of popular exercise to maintain good health, fight disease, and enhance the quality of life in China. The eighth section of Eight-section Brocade, which has been known as the tiptoe posture, may stimulate the immune system and enhance the power of osteogenesis by the perpendicular stress resulting from the body gravity. But the modified eighth section of eight-sectioned brocade has included 2 training exercise, such as the tiptoe and the swat stance. For the latter, has been recommended by American Sports Association as the position which contributes to prevent the osteoporosis. And when the heel has suddenly been put down, the gravity from the body strikes the ground, which produces the reaction propulsion, in turn, which has resulted in the vibration of the body. However, vibration exercise nowadays has been broadly available to exerciser and patient.^33^ So in fact, Our MESE has been composed of 3 kinds of exercise. So MESE exercise is the most effective for the improvement of the symptom and indexes in postmenopausal women.

## CONCLUSIONS

Our study reported greater improvement in the MESE and MESE + Ca group. We found that the treatment of MESE exercise was the most effective for alleviating the chronic pain, increasing the left femoral neck and lumbar BMD and balance in posture, which are associated with the increased bone fragility and higher risk of fracture. Importantly, the low attrition and exercise compliance coupled with the low number of adverse events indicates that this type of exercise program is safe, feasible, and well tolerated by previously untrained, but otherwise healthy older men.

Certainly, clearly more research is needed in order to better understand the specific therapeutic potential of MESE as an exercise model. Moreover, there seems to be a certain need for studies to assess any potential long-term risks. However, with appropriate introduction to the exercise modality, MESE exercise seems to be reasonably safe for most people.
